# Impact of COVID-19 anxiety on functional foods consuming intention: role of electronic word of mouth^☆^

**DOI:** 10.1016/j.heliyon.2022.e11344

**Published:** 2022-11-01

**Authors:** Thuy Thu Nguyen, Hoa Thi Thanh Phan

**Affiliations:** Department of Business Administration, National Economics University, 207 Giai Phong, Hai Ba Trung, Hanoi, Viet Nam

**Keywords:** COVID-19 anxiety, Health consciousness, eWOM communication, Trust on eWOM, Functional foods, Purchase intentions

## Abstract

**Purpose:**

The COVID-19 pandemic, which involving mass quarantines in many nations, has affected consumer health consciousness behaviors, including food consumption. This study aimed to investigate the impact of COVID-19 anxiety on functional food consumption within the framework of changes in health concerns and electronic word-of-mouth communication through digital platforms, paying special attention to the moderating effect of trust on eWOM information.

**Design:**

/methodology/approach: This cross-sectional study included 527 participants. The data were analyzed using a structural equation modeling approach. Mediating moderating effects were tested using bootstrapping and multigroup methods.

**Findings:**

Anxiety about the COVID-19 virus has a great impact on individuals’ health concerns, word-of-mouth information seeking, and functional food consumption intention. Mediating effects of health consciousness and eWOM on functional food consumption intention were also observed. The anxiety about the COVID-19 virus and health consciousness triggers eWOM communication, and is a strong driver of intention to purchase functional foods if people trust the eWOM information source.

**Originality/value:**

While scholars have demonstrated the impact of COVID-19 on human behavior, a largely uninvestigated issue is the effect of COVID-19 virus anxiety on health product consumption. This is one of the first studies to examine the interrelationships among COVID-19 anxiety, health consciousness, eWOM, and functional food consumption intention.

This study provides valuable insights into consumer behavior during the COVID-19 pandemic. Future research should consider the effect of COVID-19 virus anxiety on health-related issues and nutritional behavioral consequences. Based on the results, implications for managers and researchers are proposed.

## Highlights

• Covid-19 virus anxiety impact individuals’ concern of health and functional foods consuming intention.
• eWOM communication mediate the effect of anxiety of Covid-19 virus and functional foods consuming intention.
• Trust on eWOM information moderates these relations.

## 1 Introduction

Since its discovery in Wuhan City (China) in 2019, the COVID-19 pandemic has rapidly and profoundly affected people’s well-being and produced a global mental health crisis, especially in countries with a large number of people affected and weak healthcare systems (Shimpo et al., 2021). Continuous daily updates of information on hospitalized and death cases, compulsory or voluntary quarantines, and lockdown measures deteriorated individuals’ fear and anxiety (Wang et al., 2020). Anxiety and depression symptoms are even more prevalent in individuals after the viral infection. The literature suggests that COVID-19 survivors are at an increased risk of mood and anxiety disorders many months post-infection (Li et al., 2021). The literature highlights that pandemic anxiety creates fundamental behavioral changes in individuals’ lives, especially consumption habits (Saravanan et al., 2020; Saricali et al., 2022; Satici et al., 2021). People stock up processed foods, buy healthier products, and shop online more often (Aksoy et al., 2021; Shimpo et al., 2021). However, we do not know exactly how individual differences in proneness to anxiety play a role in different emotional reactions and consumptions (Renström and Bäck, 2021). Zwanka and Buff (2020) suggested that health-protected behaviors under the pressure of the COVID-19 anxiety need further in-depth studies. More focus is needed to understand human behaviors in the consumption of health-related products as unintended consequences of disease anxiety.

This study aimed to answer the question: Does COVID-19 anxiety impact functional food purchasing intention within the framework of interactions with health consciousness and electronic word-of-mouth communication? To our knowledge, no prior research has been conducted on the above-mentioned relationships as a result of COVID-19 anxiety.

Excessive anxiety can change individuals’ rational thinking and may trigger a new pattern of behavior (Cypryańska and Nezlek, 2020; Ilicic and Brennan, 2022). People suffering from pandemic-related anxiety tend to adopt various approaches for managing the potential risk of viral infection, including increased consumption of health products (Shimpo et al., 2021). Healthiness has become an important concern for individuals during the COVID-19 pandemic due to their desire to have a strong immune system (Zwanka and Buff, 2020). Remarkably, as an unintended consequence of pandemic fear, a shift to healthy food and nutrition consumption has also been observed (Shimpo et al., 2021; Aksoy et al., 2021). COVID-19 fear and anxiety have boosted interest in foods and nutrition that deliver well-being benefits, such as immunity empowerment and stress management (Shen et al., 2020).

Traditionally, functional foods have been considered as supplementary medicines tools and materials for preventing and treating chronic diseases (Goetzke et al., 2014). Functional foods include a variety of relevant components to improve one’s health status or reduce the risk (non-prevention) of disease beyond the traditional nutrients it would normally contain (Boludai and Capilla, 2017). Since the active components of many functional foods are derived from foods or herbal medicines, individuals believe that they can substitute functional foods and beverages for some medicines. However, consumer behavior concerning functional product consumption as a consequence of COVID-19 anxiety has been neglected (Aksoy, 2021).

Additionally, it has been demonstrated that being health-conscious in the emerging uncertainty, individuals immediately started searching for nutrition-based treatment information (Soroya, 2021). The recent and rapid growth of Internet technology, as well as social distance law in almost all countries due to the pandemic, has dramatically changed the parameters of consumer behaviors (Bambauer and Mangold, 2011). Consumers during the pandemic have widely used online tools to search for product information. They use various media (radio, TV, electronic newspapers, and magazines), among which electronic word-of-mouth (eWOM) information is prominent. Consumers search for information through social media (Facebook, Twitter, Zalo, etc.) and search engines, such as Google, and scientific and official websites, to make themselves comfortable and informative before purchasing services or products (Chen et al., 2016). EWOM has become a key channel for providing pre-purchase information for product consumption (Chang et al., 2005). In this study, we are interested in the influence of feelings of COVID-19 anxiety on eWOM communication and argue that not only do eWOM information sources play a role, but also that perceived trust in eWOM information can moderate the relationship between COVID-19 anxiety and functional product consumption intention.

**Figure 1:**
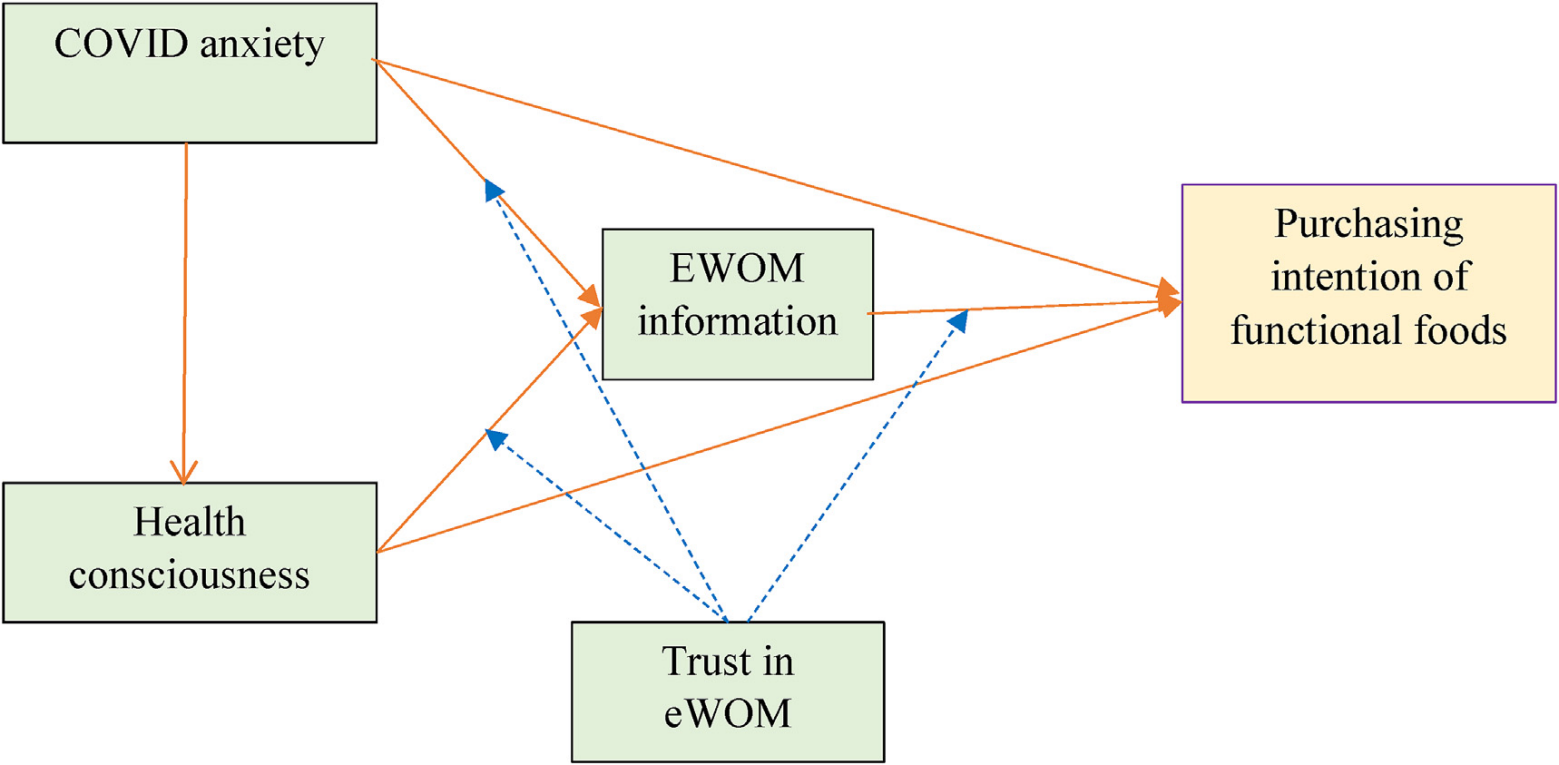
Research model.

This study had three main goals: (1) to test the impact of COVID anxiety on health consciousness and purchasing intention of functional foods; (2) to test the mediating effect of eWOM communication on the relationship between COVID anxiety, health consciousness, and purchasing intention of functional foods; and (3) to examine the moderating effect of trust on eWOM information on the relationships between variables in the structural model (Figure 1). The results of this study are expected to contribute to the literature by enhancing our understanding of the impacts of COVID-19 anxiety on individuals’ consumption behaviors.

## 2 Literature review and research hypothesis

### 2.1 The effect of COVID-19 anxiety

Anxiety is a cognitive-emotional process described as worry about future events. Anxiety is elicited when the threat is ambiguous, uncertain, or less specified, and the reaction is a prolonged emotional state (Saravanan et al., 2020).

Previous studies have recognized the effect of the coronavirus disease 2019, not only on physical health problems, but also on mental health disorders (Lee et al., 2020; Saricali et al., 2022; Le et al., 2020). Since COVID-19 is identified as a deadly disease, during the pandemic, people are anxious about severe complications caused by the disease and worry that they might have to be isolated after contracting the disease, or lose loved ones to the disease (Wang et al., 2020). The massive impact of COVID-19 on human life and health has led to fear and anxiety. Continuous information about the pandemic and a focus on the spread and danger of the virus also evoke anxiety. Viral anxiety appears to be the main psychological symptom associated with COVID-19 (Lee et al., 2020). A wide literature base has shown that pandemic-related psychological distress tends to lead to elevated levels of health anxiety, which may last well beyond the course of the pandemic (Duong, 2021).

Higher levels of anxiety or worry may be associated with an increase in behavioral changes. COVID-19 anxiety can be seen as a stressor that elicits a strong response among people (Saricali et al., 2022; Wang et al., 2020). COVID-19 anxiety led people to take actions aimed at planning behaviors to anticipate and avoid future harm, especially health-protecting behaviors (Cypryańska and Nezlek, 2020). Under panic emotional conditions, viral anxiety directs people to be more concerned about their personal health, and health has become the top priority (Pu et al., 2021). Previous studies have reported a high sensitivity of health consciousness to fear, stress, and emotional factors (Soroya et al., 2021). Daily information about the increasing number of deaths and severe long-term illnesses suffered by those who have tested positive for the coronavirus is making people more health-conscious. They are willing to undertake healthcare behaviors and integrate health concerns into their daily activities (Pu et al., 2021).

Hypothesis 1 COVID-19 virus anxiety has a positive impact on health consciousness

Previous research indicate that anxiety evokes uncertainty, increases perceived risks, and causes feelings of lack of control (Saricali et al., 2022). A wide body of experimental and epidemiological studies has shown that the higher the perceived level of risk, the greater the intention to take action to relieve it (Cypryańska and Nezlek, 2020). COVID-19 anxiety has been reported to result in health behaviors, including diet and healthy consumption, as a precautionary plan for adverse risks (Shimpo et al., 2021; Shen et al., 2020). The availability of COVID-19 vaccines and antiviral medication to prevent and control the virus faces continuous challenges in terms of vaccine sharing and medicine accessibility in low- and middle-income countries (Le et al., 2020). While vaccines are not available, people try to reduce pandemic risks by consuming and supplementing adequate functional foods. Functional food is regarded as a special kind of food that has been satisfactorily demonstrated to beneficially affect one or more target functions in the body. Beyond its adequate nutritional effects, functional food reduces the risk of disease and/or improves the state of health and well-being (Boluda and Capilla, 2017). The health of the immune system is the top priority motivating the use of supplements and functional foods, with probiotics being among the most sought-after ingredients consumers are looking to increase in their diet (Chen, 2011; Goetzke et al., 2014). Functional foods optimize the immune system’s capacity to prevent and control pathogenic viral infections, whereas physical activity augments such protective benefits (Aksoy et al., 2021). People believe that healthy foods can help maintain optimal levels in the human body, enhance the immune system, and consequently provide important prevention against COVID-19 (Shimpo et al., 2021).

Hypothesis 2 COVID-19 virus anxiety has a positive impact on the purchase intention of functional foods

### 2.2 The effect of health consciousness

Health consciousness is the degree to which individuals care about their health or the degree to which a person is inclined to take health-related actions (Pu et al., 2021). In recent years, scholars have focused on the influence of health consciousness on various health behaviors, including healthy consumption. The more health-conscious people are, the more likely they are to have healthy habits (Aksoy et al., 2021; Pu et al., 2021). Health consciousness guides people to put forth efforts to maintain a healthy life and engage in healthy behaviors (Goetzke et al., 2014). Functional foods are believed to decrease the risk of several diseases and promote health benefits in several essential parts of human physiology. Health consciousness makes consumers consider buying a product that helps them stay safe and healthy; therefore, they may be more seriously concerned about functional food products than consumers with low health consciousness (Kim and Chung, 2011). Since consumers are more concerned about their health, and their perception that diet directly affects healthiness is spreading, functional foods have played a fundamental role in healthy habits. According to Boluda and Capilla (2017), health is one of the central variables in the study of consumer behavior toward functional foods. Nguyen et al. (2019) demonstrated that consumers with high health consciousness exhibit a more positive attitude and intention toward functional foods.

Hypothesis 3 Health consciousness has a positive impact on the purchase intention of functional foods

### 2.3 The role of EWOM communication

The literature has identified electronic word of mouth (eWOM), or "between–customer communication" as a probable driver of consumer decision-making. EWOM is regarded as a negative or positive statement made by potential, actual, or former customers about a company or product that is made available to the society via the Internet (Bambauer and Mangold, 2011). Consumers look for eWOM information before purchasing a product, and 80 percent of consumers consult online customer reviews before making purchase decisions (Mafe et al., 2020; Chang et al., 2005; Cheung and Thadani, 2012).

The fear accompanying COVID-19 will engage consumers in seeking information and obtaining vital information on their products of interest. Obtaining and updating information to fill this information gap is one of the basic motivations for human information-seeking behavior. Consumers need information to control, avoid, deal with, or respond to fear and other perceived risk (Chen et al., 2016). Moreover, COVID-19 social distancing regulations lead to a high level of customer interactivity and social presence in e-commerce platforms (Ardyan et al., 2021; Zwanka and Buff, 2020). As a result of long-lasting quarantine time, people are more attentive to online social communication and online shopping (Aksoy et al., 2021; Shimpo et al., 2021). Consumers exacerbate online eWOM communication (Cheung and Thadani, 2012). Goetzke et al. (2014), Soroya et al. (2021) found a reciprocal relationship between health anxiety and online health-related product information communications.

Moreover, previous research reported that consumers follow others when making a purchase decision (Bambauer and Mangold, 2011). Consumers may choose to buy the same brand or product as a safe way to avoid uncertainty or risk (Chen et al., 2016). Chang et al. (2005) indicated that with the development of social media, consumers have a tendency to depend on online reviews or inquiries before making purchase decisions, especially in uncertain situations. With the rapid development of the functional food industry, new food technologies and ingredients that are novel to consumers are being continuously applied in functional food production (Goetzke et al., 2014). Consumers who commonly lack relevant professional knowledge of functional foods must rely on social information sources when making decisions to reduce their uncertainty. EWOM is a preferred source of social information to consult because, as Chen et al. (2016) indicated, customer-to-customer information is less susceptible to bias from commercial motives; therefore, it is considered more credible than enterprise information. EWOM, in the form of customer-to-customer reviews and exchanges of information, affects the overall perceptions of the value of a product, service, or brand (Cheung and Thadani, 2012). eWOM information provides consumers with a sense of security when making buying decisions (Yusuf et al., 2018). Huang et al. (2020) showed that the more people are interested in health, the more frequently they look for information on functional foods. Information about a product from eWOM is expected to create awareness, knowledge, and form or change an individual’s purchase intention. Therefore, we propose that health concerns and virus anxiety engage people in eWOM communication, and eWOM communication as a platform leads to the intention to purchase functional foods. EWOM information mediates the relationship between COVID-19 virus anxiety, and health consciousness and purchase intention of functional foods.

Hypothesis 4a COVID-19 virus anxiety has a positive impact on EWOM Information

Hypothesis 4b Health consciousness has a positive impact on EWOM Information

Hypothesis 4c EWOM Information has a positive impact on the purchase intention of functional foods

### 2.4 The moderator effect of trust on eWOM information

The literature has recognized two online eWOM features that influence consumer decisions (Mafe et al., 2020). These include a variety of eWOM information and source credibility. We predict that not only eWOM communication can impact purchase intention, but trust in eWOM information can also effectively influence purchase intention. Ewom provides a massive amount of information to the consumers because of concerns about credibility and authenticity leading to confusion among the consumers (Chen et al., 2019). The mixed content available from online eWOM information sources may amplify risk perceptions due to information overload (Filieri et al., 2015). Literature also shows that information overload, in general, adversely affects human information processing capacity and actions (Ardyan et al., 2021). Another negative outcome of eWOM communication is that consumers cannot discern between real and fake news or negative and positive reviews (Chen et al., 2016). According to Yusuf et al. (2018), judging of the eWOM information credibility is a decisive factor in the information persuasion process. eWOM is different from face-to-face communication; in eWOM, consumers exchange or share information with unknown people on the Internet with no prior relationship. Therefore, if consumers believe that eWOM information is credible, they gain more confidence in adopting eWOM information and rely on it when thinking about purchasing (Abedi et al., 2019). The judgment of the credibility of information comes from the credibility of the reviewers or the credibility of the information channel, which determines whether potential customers learn and adopt, believe, and follow eWOM information (Filieri et al., 2015). Source trustworthiness and credibility are considered key predictors of consumers' acceptance of traditional WOM (Bambauer and Mangold, 2011). Relationships between customers’ trust in eWOM information, purchase attitude, and intention have been found in e-commerce studies (Yusuf et al., 2018; Roudposhti et al., 2018). Filieri et al. (2015) found that the source trustworthiness of eWOM information increases the awareness of consumers’ nutritional knowledge, makes customers familiar with and interested in functional foods, and subsequently affects purchase intention. Huang et al. (2020) indicated that consumers would believe in functional foods if they trust the information channels.

Hypothesis 5a Trust of eWOM information moderate the impact of COVID-19 virus anxiety on eWOM information

Hypothesis 5b Trust of eWOM information moderate the impact of health consciousness on eWOM information

Hypothesis 5c Trust of eWOM information moderate the impact of eWOM information on purchase intention of functional foods

## 3 Materials and methods

### 3.1 Questionnaire design

All the scales of the model were adapted from the existing literature and measured using 5-point Likert scale from (1) “strongly disagree” to (5) “strongly agree”. Initially, the questionnaire was translated from English to Vietnamese and later translated back into English with the help of a native English translator, and then pre-tested with a qualitative survey for the accuracy of meaning and translation following the scale adaption protocol detailed by Duong et al. (2021).

Coronavirus anxiety was measured using the English version of the scale published by Lee et al. (2020) following the scale adaption protocol as detailed in Raggiotto et al. (2020). This scale assesses the physiologically based symptoms associated with COVID-19 anxiety. The scale loads on a single factor (e.g., “I felt dizzy, lightheaded, or fainted, when I read or listened to news about the Coronavirus”). The measure was scored using a 5-point Likert scale (0 = not at all to 4 = nearly every day over the last 2 weeks). The participants were asked to rate how frequently they experienced each anxiety symptom. Lower scores indicated lower levels of viral anxiety. The Coronavirus Anxiety Scale has demonstrated good validity and reliability in recent studies (Duong, 2021; Lee et al., 2020). In this study, the scale had a Cronbach α = 0.859.

Purchase intention (PI) was measured using four items adapted from Kim and Chung (2011). The instrument has convergent validity and is considered a reliable measure of consuming intention (e.g., “It is likely that I will purchase functional foods,” “If functional food is available, I buy it”). In this study, the scale had a Cronbach α = 0.848.

Health consciousness (HC) was assessed using five items adapted from Chen (2011). This scale is commonly used as a measure of health consciousness and is considered reliable and valid (Huang et al. (2020). In this study, the HC group had a Cronbach α 0.789.

The eWOM communication (WI) construct was measured using a four-item scale adapted from Bambauer and Mangold (2011). In this study, the scale had a Cronbach α 0.912. Trust in eWOM Information credibility (TW) was measured using a four-item scale adapted from Filieri et al. (2015). In this study, the scale had a Cronbach α = 0.876.

The questionnaire also included demographic information on the participants: age, marital status, gender, occupational status, educational status, income, and current place of residence.

### 3.2 Data collection

The sample comprised 527 responses. Since a safe distance should be maintained between people during the COVID-19 pandemic induced lockdown, questionnaires could not be collected offline. Data were collected via self-administered online surveys using a convenience sampling technique. The official study was conducted in June 2021 by sending soft electronic copies of the survey questionnaire online via Google docs to about 800 email addresses. Also, questionnaire were uploaded to various groups’, associations’, and social Internet networks (Facebook and Zalo groups). Participation was voluntary and anonymous. A total of 527 responses were received. Among these 58.4% were female and 41.6% were male respondents. 52.9% of the respondents were less than 25 years, and 19.3% were between 26-35 years, and 9.2% were above 55 years old. 73.4% of the respondents were single, 51.6% had bachelor or college degrees 39.6% were students, 33.3% were workers and freelancers, and 27% had retired. Also, 25.8% respondents received incomes of over 800 USD, 39.5% received less than 300 USD, 17.1% received between 500–800 USD, and 17.6% received between 300–500 USD per month. More importantly, a majority of the respondents experienced using functional foods (72.3%) and only 27.7% had never used any functional foods.

### 3.3 Methodology

As all data were self-reported by the respondents, to avoid potential common method bias, we informed the participants that their responses would be kept confidential and used only for scientific research. The questionnaires were answered anonymously. We then conducted a Harman's one-factor test for statistical control. All items were analyzed using unrotated principal component factor analysis (Podsakoff et al., 2003). The results identified five factors with eigenvalues greater than 1. The variance explained by the first factor was only 18.75% under a critical value of 40%. This result confirmed the absence of any serious common method bias in the study.

SPSS version 23 was used for initial analyses and data exploration to confirm the normality of the distributions and test the reliability and validity of the measurements. Confirmatory factor analysis (CFA) using AMOS software version 23 was used to test the measurements. This study used Structural Equation Modeling, which is regarded as a statistically effective and powerful approach to examine structural invariance and recommended 5,000 bootstraps to directly test the significance of mediation effects (Hair et al., 2014). The bootstrapping method has been found to be a more accurate test of mediation effects than other available tools (Preacher and Hayes, 2008). Furthermore, a multi-group analysis was performed to investigate the moderating effect of perceived trust on eWOM in the structural model. We used the median split approach to divide the sample into two sub-groups: high and low trust perception. We conducted a chi-square difference test to compare the unconstrained model (all paths were not constrained across the two subgroups) and constrained model (all paths were restricted across the two subgroups). A moderating effect exists if the constrained model presents a higher chi-square value than that of the unconstrained model.

## 4 Results and discussion

### 4.1 Reliability and validity analyses

Skewness and kurtosis methods were used to test the univariate normality of the scales. All items had a skewness value lower than 1 and a kurtosis value lower than 8 (Table 1). The results indicated a normal distribution for these scales (Hair et al., 2014). The reliability test with Cronbach’s alpha analysis of each variable shows that in all cases, Cronbach’s alpha exceeded 0.70. All items have a value of Cronbach's alpha if the item deleted for each item is lower than the scale‘s Cronbach’s alpha. Thus, all constructs met acceptable values for the criteria of composite reliability.

**Table 1:** Demographic statistics.

Demographic statistics (527)	Frequency (persons)	Percentage (%)
Gender		
Male	219	41.6
Female	308	58.4
Age		
16–25	279	52.9
26–35	102	19.3
36–55	98	18.6
Over 55	48	9.2
Marital status		
Single	387	73.4
Others	140	26.3
Education		
Undergraduate	229	43.5
Bachelor’s degree	161	30.6
postgraduate degree	131	25.9
Employment status		
Student	209	39.6
Still working	175	33.3
Retired	143	27.1
Household monthly income		
Under 300 $	208	39.5
From 300$ to 500&	93	17.6
From 500$ to 800$	90	17.1
Above 800$	136	25.8
Experience of using functional foods?		
Yes	381	72.3
No	146	27.7

(Source: authors’ survey).

The KMO – Bartlett’s test showed a KMO value of 0.866, a cumulative total variance of 68.35%, and a Bartlett’s value of 0.000, showing a high sensitivity of the test. All the reflective construct loadings had a minimum of 0.657 reference value, and no item had higher loadings in other constructs, different from the original scale (Table 2). These scales are considered to have good validity (Hair et al., 2014). As a result, the consistency and internal reliability of the scale were confirmed.

**Table 2:** Descriptive Statistics and Results of exploratory factor analysis.

Scale	Factor Loading						Descriptive Statistics					
	Component						Mean Statistic	Std. Deviation Statistic	Skewness Statistic	Std. Error	Kurtosis = Statistic	Std. Error
		1	2	3	4	5						
Health consciousness	HC1				.755		3.691	.9069	-.409	.106	.042	.212
	HC2				.657		3.345	1.0619	-.102	.106	-.675	.212
	HC3				.711		3.571	.9363	-.235	.106	-.400	.212
	HC4				.761		4.101	.8474	-.870	.106	.801	.212
	HC5				.778		3.962	.8821	-.543	.106	-.181	.212
Virus anxiety	CA1	.882					3.250	1.0473	-.106	.106	-.507	.212
	CA2	.816					3.226	1.0338	-.152	.106	-.325	.212
	CA3	.848					3.366	1.0098	-.226	.106	-.382	.212
	CA4	.702					3.510	1.0260	-.277	.106	-.494	.212
	CA5	.699					3.573	1.0290	-.334	.106	-.484	.212
Purchase intention	INT1					.839	3.602	1.0098	-.340	.106	-.318	.212
	INT2					.857	3.319	.9376	-.174	.106	.059	.212
	INT3					.791	3.171	1.0269	-.114	.106	-.297	.212
	INT4					.800	3.653	.9627	-.375	.106	-.260	.212
eWOM information	EWOM1		.827				3.353	1.0860	-.539	.106	-.356	.212
	EWOM2		.924				3.486	1.1130	-.620	.106	-.298	.212
	EWOM3		.914				3.444	1.1236	-.542	.106	-.362	.212
	EWOM4		.843				3.342	1.1324	-.502	.106	-.380	.212
Trust in eWOM	TM1			.729			3.034	1.0404	-.150	.106	-.414	.212
	TM2			.909			2.825	1.0169	-.037	.106	-.403	.212
	TM3			.824			3.034	.9507	-.122	.106	-.135	.212
	TM4			.870			2.954	.9855	-.040	.106	-.209	.212

(Source: authors’ survey).

Then, CFA is used to test the assessment of fit between the observed data and prior conceptualized constructs, and the results show that the goodness of fit is acceptable. The proposed model had a CMIN/df value of 2.594, goodness of fit index (GFI) of 0.935, comparative fit index (CFI) of 0.959, TLI of 0.951, NFI of 0.935, and IFI of 0.959, all higher than the benchmark value of 0.90. RMSEA = 0.055 is acceptable. Thus, the results exhibit adequate convergent validity and fit (Hu and Bentler, 1998). All CR values of the latent factors in this model were above the cutoff value of 0.7 and the variance extraction was greater than 0.50. The results indicate that all constructs of the measurement model adequately demonstrated reliability and convergent validity. Discriminate validity was examined using average variance extraction (AVE). Although the AVE value of health consciousness is only 0.414, Malhotra and Dash (2011) argue that AVE is often too strict and reliability can be established through CR alone; thus, this could be accepted. The AVE values of the three other scales were all above the cutoff value of 0.50 and the root square of each construct’s AVE value was higher than their MSV, ensuring the constructs’ discriminant validity (Table 3).

**Table 3:** Reliability and validity of measurements.

	CR	AVE	MSV	MaxR(H)	CA	EWOM	HC	PI
CA	0.849	0.540	0.330	0.895	0.735			
EWOM	0.914	0.726	0.126	0.924	0.253∗∗∗	0.852		
HC	0.777	0.413	0.091	0.785	0.270∗∗∗	0.069	0.643	
PI	0.853	0.594	0.330	0.865	0.629∗∗∗	0.440∗∗∗	0.261∗∗∗	0.768

CA = COVID-19 Virus anxiety, PI = Purchase intention, HC = Health consciousness.

(Source: authors’ survey).

### 4.2 Hypothesis testing

We performed Structural Equation Modeling analysis, followed by a bias-corrected bootstrapping run at 5,000 sub-samples to test the hypothesized path model. The Structural Equation Modeling path analysis results and the hypothesis testing results are summarized in Table 4. As recommended by Preacher and Hayes (2008), RMSEA values smaller than 0.08 and the CFI, GFI, and TLI values greater than 0.9 indicate an acceptable fit (Hu and Bentler, 1998).

**Table 4:** Summary of hypotheses tests.

Hypothesis				Estimate	S.E.	C.R.	P	Outcome
H1	HC	<---	CA	.194	.038	5.102	∗∗∗	Support
H4a	EWOM	<---	CA	.238	.047	5.030	∗∗∗	Support
H4b	EWOM	<---	HC	.000	.069	.000	0.989	Reject
H4c	PI	<---	EWOM	.217	.042	5.196	∗∗∗	Support
H2	PI	<---	CA	.431	.044	9.820	∗∗∗	Support
H3	PI	<---	HC	.200	.060	3.331	∗∗∗	Support

(Source: authors’ survey).

COVID-19 anxiety perceptions were found to be directly and positively related to functional food purchasing behaviors (β = 0.471, p < 0.001), health consciousness (β = 0.94, p < 0.001), and eWOM (β = 0.238, p < 0.001). The path linking health consciousness to purchase intention was also found to be significantly positive (β = 0.123, p < 0.05). Hypotheses H1, H2, and H3 are supported by the research data (Table 4). The path linking health consciousness to eWOM is not found to be significantly positive (p > 0.1), so H4b is not supported by the research data.

The bootstrapping method was used to test the significance of the mediating effect with a 95% biased-corrected confidence interval and 5000 bootstrap samples. The assessments of the total, direct, and indirect effects are shown in Table 5. The results indicated that the total effect of virus anxiety on functional food purchasing behaviors was significant (standardized β = 0.574, two-tailed significance <0.001). The direct effect was significant (standardized β = 0.475, two-tailed significance p < 0.001). The indirect effect was 0.100, which was statistically different from zero (two-tailed significance, p < 0.001). The findings validated the direct and indirect impacts of virus anxiety on purchase intention. Our statistics suggest that EWOM partly mediates the relationship between virus anxiety and functional food purchasing behaviors (P < 0.001). This implies that COVID-19 virus anxiety leads to more health concerns and more engagement in seeking eWOM information and communication, and health concerns and eWOM as a platform impact functional food purchasing intention. This result contradicts the positive, direct effect of health consciousness on eWOM. The total effect of health consciousness on functional food purchase intention was also not significant (two-tailed significance, p > 0.001). Therefore, eWOM does not mediate the relationship between health consciousness and functional food-purchasing intentions.

**Table 5:** Mediation effects with bootstrapping test (Two Tailed Significance 95%).

Path		CA- HC	CA- EW	CA- PI	HC- EW	HC- PI	EW- PI
**Direct effect**	Standardized Estimate	.271	.253	.475	.000	.158	.224
	*Two Tailed Significance*	*.000*	*.000*	*.000*	*.994*	*.046*	*.000*
**Indirect effect**	Standardized Estimate			.100			
	*Two Tailed Significance*			*.000*			
**Total effect**	Standardized Estimate	.271	.253	.574	.000	.158	.224
	*Two Tailed Significance*	*.000*	*.000*	*.000*	*.994*	*.001*	*.000*

CA = COVID-19 Virus anxiety, PI = Purchase intention, HC = Health consciousness.

(Source: our own study).

The sample was divided into two sub-groups of high and low trust perceptions using the median split method. The first group, who had high trust in the eWOM information channel, included 226 respondents (42.9%), 55.7% of whom were female, 54% were aged under 35, 44.2% had a university degree, and 72.6% were single. The second group, the low trust group, included 301 people (57.1%), 58.8% of whom were female, 50.8% were aged under 35, 42.9.2% had a university degree, and 40.2% had less than 300 USD income per month. The chi-square statistic demonstrates that the constrained (χ 2 = 524.631, df = 260) and unconstrained models (χ 2 = 503.980, df = 254) were significantly different (Δχ 2 = 20.651, Δdf = 6, p < 0.005), supporting the moderating effect of perceived trust on eWOM information on structural relationships (Table 6).

**Table 6:** Moderating effect of trust in eWOM information - non-standardized estimates.

Hypothesis				Standardized Coefficients		χ 2 (d.f.)	Δχ^2^ (Δd.f. = 1)
				High trust (n = 226)	Low trust (n = 301)		
Constrained Model						524.631 (260)	
H1	HC	<--	CA	.294∗∗∗	.293∗∗∗	524.355 (259)	0.276
H4a	EWOM	<--	CA	.280∗∗∗	.087	519.003 (259)	5.628∗∗
H4b	EWOM	<--	HC	.248∗∗∗	-.119	512.114 (259)	12.517∗∗∗
H4c	PI	<--	EWOM	.195∗∗	.133∗	517.995 (259)	3.282∗
H2	PI	<--	CA	.556∗∗∗	.335∗∗∗	520.184 (259)	4.447∗∗
H3	PI	<--	HC	.174∗∗	.192∗∗	524.590 (259)	0.041

∗∗∗, ∗∗, ∗ 1%, 5%, 10% significance levels, respectively (Source: authors’ survey).

To accurately detect the moderating effects of perceived trust on eWOM information on specific paths in the proposed model, a battery of chi-square difference tests was applied to compare the constrained models with seven diverse models separately, each retaining only one of the structural paths to be freely estimated. As illustrated in Table 6, perceived trust on eWOM information moderated four of the seven structural relationships. Specifically, the effect of CA on EWOM communication was significant for the high-trust group (β = 0. 280, p < 0.001) and not significant in the low-trust group (β = 0.087, p > 0.01). The effect of eWOM communication on purchase intention was stronger in the high-trust group (β = 0.195, p < 0.005) than in the low-trust group (β = 0.133, p < 0.1). The impact of health consciousness on eWOM communication was significant in the high-trust group (β = 0. 248, p < 0.001) and was not significant in the low-trust group (p > 0.1). Moreover, students with high trust on eWOM perception (β = 0.556, p < 0.001) exhibited a larger path effect than students with low trust perception (β = 0.335, p < 0.001) in the influence of CA on purchase intention. Thus, all the hypotheses of moderating effects H5a, H5b, and H5c are supported.

### 4.3 Discussions

This is the first study to investigate the impact of the anxiety of COVID-19 on functional food purchasing intention, taking into consideration the interrelationship between health consciousness, eWOM communication, and eWOM information creativity. Little research has been conducted on the impact of COVID-19 anxiety on the consumption of health products. This study shows that individuals with COVID-19 anxiety take precautionary actions to reduce their risk, are more concerned about health, actively obtain information from eWOM, and promote healthy consumption of functional foods.

This study verified the significant influence of health-related anxiety on the intention to purchase functional foods. This is in line with the latest studies by Cypryańska and Nezlek (2020), Saravanan et al. (2020), and Shen et al. (2020), who insisted that the anxiety of the COVID-19 pandemic could shift consumers’ behavior in a more sustainable and healthier direction. This result reflects the popular belief that functional foods are an effective tool for reducing the chances of exposure to respiratory viruses and boosting human immunity in the event of exposure (Aksoy et al., 2021; Boluda and Capilla, 2017).

People with higher levels of viral anxiety are more likely to show higher levels of health concerns. Our findings regarding the behavioral consequences of COVID-19 anxiety were similar to those found by others in prior studies of CypryańskaI and Nezlek (2020). That is, in the face of a dangerous virus, the more anxious the people are, the more likely they are to be concerned about their health and ready to take action to protect their health. With the spread of the news relating to the COVID-19 coronavirus disease, the COVID-19 anxiety has made people pay more attention to personal health problems and people’s health consciousness has greatly increased (Aksoy et al., 2021; Shimpo et al., 2020). The support for Hypothesis 2 also validated our argument that health consciousness is positively related to the intention to purchase healthy products. The positive correlation between health consciousness and functional food purchase intention (standardized β=0.174) is consistent with the arguments of Nguyen et al. (2019), Boluda and Capilla (2017), and Kim and Chung (2011) that health concern is one of the key variables in research on functional food consumer behaviors. A higher degree of health care reflects a more positive intention to buy functional foods. This result supports the literature that health-conscious consumers are interested in healthy and nutrition food (Aksoy et al., 2021; Landström et al., 2007). This study confirms the positive effect of health consciousness on health product consumption, in line with Landström et al. (2007) and Pu et al. (2021). Consequently, the findings confirmed the partial mediating effect of health consciousness on the relationship between COVID-19 anxiety and functional food purchasing intention. The results report that COVID-19 anxiety increases functional product purchasing intention, partly because of health safety concerns. These findings support previous theoretical models that insist that health-related motivation to prevent disease or improve health is considered the primary cause of health behavior (Cypryańska and Nezlek, 2020; Landström et al., 2007).

This result implies that virus anxiety changes individual behaviors in interpersonal and social communication and information seeking toward more usage of eWOM information. These results are in agreement with studies in the current review, which found evidence to suggest that shoppers are motivated to use electronic information sources because of their convenience and ability to save time, avoid crowds, and multitask while shopping in a covid lockdown period (Friesen, 2020, Le et al., 2020). Anxiety and fear of the viral pandemic can motivate individuals to seek information for decision-making by looking for information on healthy foods on the web to keep themselves informed of the optimization of nutrient intake through well-balanced meals and the use of good practices in food selection and conservation to address adverse COVID-19 health risks (Soroya et al., 2021). Huang et al. (2020) mentioned the role of eWOM communication, including social media, information seeking on the web, and online social networking during the COVID pandemic. Although consumers consult a variety of information sources, this study emphasizes that eWOM remains an important source of information during a pandemic.

This research study is also consistent with previous marketing literature, where eWOM is an essential ingredient in promoting purchase intention. When consumers exhibit a strong affinity for engaging in eWOM, they tend to have greater purchase intentions (Huang et al., 2020; Shen et al., 2020). This power interpersonal influence through eWOM communication has been well recognized in consumer literature. The findings of Chen et al. (2016), Cheung and Thadani (2012), and Abedi et al. (2019) reported that electronic word of mouth in the form of user reviews, testimonials, descriptive sentences or articles written by customers, helpfulness rating, or electronic comments on the Internet can help customers gain confidence and conformity of the product, help customers set their expectations and attitudes before making a purchase decision, thus increase their purchase intention. These findings provide empirical evidence supporting the proposal that consumers are more likely to rely on eWOM sources before shopping; they use social information when making decisions, especially in uncertain situations (Ardyan et al., 2021). These results also provided a strong motivation for companies to improve eWOM activities on their shopping websites and the Internet, which can educate and provide their customers with positive information in order to change customers’ perceptions and attitudes toward the usefulness of foreign functional foods. However, we did not observe a positive effect of health consciousness on eWOM communication of functional foods, and the mediating role of eWOM in the relationship between health consciousness and functional food purchase intention was not confirmed. One reason may be the availability of medicines and proven treatments for common health diseases, as well as the availability of various health-related information sources for a common illness that will not increase eWOM communication. This requires further in-depth studies.

The research findings validated the role of trust in eWOM information in the interrelationship between COVID-19 anxiety, health consciousness, eWOM communication, and functional food purchase intention. These findings are consistent with those of previous studies, in which information credibility is crucial to information usefulness (Abedi et al., 2019; Chang et al., 2005; Scarpi et al., 2019; Yusuf et al., 2018). The general public has been bombarded with a vast array of nutritional information from various sources; consequently, trustworthy beliefs in eWOM information among consumers is a decisive factor controlling which information sources are used. When consumers consider an information to be credible, they tend to engage in eWOM (e.g., sharing or seeking information). This is in line with Cheung and Thadani (2012), Huang et al. (2020), and Filieri et al. (2015), who argue that if consumers perceive that eWOM posted on the social media platform is credible, the recommendations or reviews from others as believable or true, consumers will consider them useful and use it. Firms will have to encourage customers to attach vital clues when sharing their experiences to help other consumers establish the credibility of such messages. Functional food marketing managers should establish discussion forums, place more emphasis on social networking sites, and use famous and professional users’ reviews to attract and gain the credibility of potential customers to increase the level of information credibility, which can influence the purchase decision.

## 5 Conclusion

Coronavirus is a highly infectious and potentially lethal virus that has caused an unprecedented pandemic. This pandemic presents many psychological and behavioral challenges for individuals, including fear and anxiety. COVID-19 anxiety has boosted interest in functional foods, which are believed to deliver well-being benefits, such as improving immunity and stress management. Despite intense efforts by scholars to investigate the COVID-19 pandemic consequences, the literature remains scarce. This study is an initial effort to propose a conceptual framework for identifying the impact of COVID-19 anxiety on the intention to purchase functional food products in emerging markets. The findings of this study increase the understanding of the association between virus anxiety, health consciousness, eWOM information adoption and credibility and consumers’ functional food purchase intention. The results revealed a positive influence of COVID-19 anxiety on health consciousness, eWOM information, and purchasing intention of functional food products. Health consciousness and eWOM information partly mediated the relationship between COVID-19 anxiety and functional food purchasing intention. Trust on eWOM moderates the structural link between COVID-19 anxiety, health consciousness, and eWOM information as well as eWOM information and functional food purchasing intention. Although being more concerned about health in the context of the COVID-19 pandemic does not significantly motivate people to seek eWOM information about functional foods, it does positively impact consumers’ purchase intentions for functional food products.

This study has some limitations. First, the cross-sectional nature of this research precludes us from drawing causal relationships between COVID-19 anxiety and its long-term behavioral consequences, and a longitudinal study would be necessary to identify the relationship between the factors in this research and actual buying behaviors. Second, it would also be desirable to compare the COVID-19 anxiety effects in other countries with different social, institutional, and economic conditions. Future research should conduct cross-cultural studies comparing collectivistic and individualistic countries, North American, Western European vs. Asian countries, or developed versus developing countries. Third, this study used a convenient sampling technique, which may undermine the generalization of the results. Fourth, mental health is a complex concept that is influenced by numerous factors. Future research could further explore the interrelationship between the determinants and consequences of viral anxiety using moderated mediation models.

## Declarations

### Author contribution statement

Thuy Nguyen: Conceived and designed the experiments; Performed the experiments; Analyzed and interpreted the data; Contributed reagents, materials, analysis tools or data; Wrote the paper.

Hoa Phan: Contributed reagents, materials, analysis tools or data.

### Funding statement

This research is funded by National Economics University, Hanoi, Vietnam, No CBQT1.2021.23.

### Data availability statement

Data is available upon request.

### Declaration of interest’s statement

The authors declare no conflict of interest.

### Additional information

No additional information is available for this paper.
